# Editorial: Recent advances and future perspective in probiotics isolated from fermented foods: From quality assessment to novel products

**DOI:** 10.3389/fmicb.2023.1150175

**Published:** 2023-02-07

**Authors:** Olga S. Papadopoulou, Agapi Doulgeraki, Efstathios Panagou, Anthoula A. Argyri

**Affiliations:** ^1^Institute of Technology of Agricultural Products, Hellenic Agricultural Organization—DIMITRA, Lykovrysi, Greece; ^2^Laboratory of Microbiology and Biotechnology of Foods, Department of Food Science and Human Nutrition, School of Food and Nutritional Sciences, Agricultural University of Athens, Athens, Greece

**Keywords:** probiotics, beneficial microorganisms, fermentation, dairy and non-dairy products, quality, safety, consumer studies, metabolomics

Probiotics have gained significant importance in the last few decades and the concept of probiotics is currently well-established for consumers and the research community. The term “probiotics” is extremely broad since it encompasses a variety of microorganisms with a range of beneficial effects after human consumption i.e., improving the balance of the gut microbiota alleviate gastrointestinal infections, inhibiting the growth of pathogenic bacteria, strengthening the barrier function of the gut, improving immunity, assimilation of serum cholesterol, prevention of irritable bowel syndrome, reducing hypertension, preventing diarrhea, improvement of mental health etc. (Soomro et al., 2001). The use of probiotics in food fermentation, with the main goals of improved food preservation, distinctive sensory characteristics, and safety aspects is targeting to novel products with high added value (Papadopoulou et al., 2018). A number of studies on fermented foods have led to the conclusion that the health-promoting substances are associated with the complex microbial communities that contribute to the fermentation of these foods, as well as the substances they produce, and their consumption has long been associated to health benefits. Probiotic strains are incorporated in products such as fermented milk, yogurt, cheese, dairy desserts, and ice-cream (Espitia et al., 2016). Consequently, the dairy industry has been using fermented products as carriers of probiotic strains and the research into these products has increased. However, lately there is a growing demand for non-dairy functional foods and beverages, as dairy alternatives, driven by the increasing number of individuals with lactose intolerance and/or allergic to cow's milk protein, with high cholesterol levels, or vegans. The idea of this Research Topic was to provide scientific knowledge and updated information related to probiotics and their application in the production, processing and preservation of the new food products. This topic included four studies i.e., three research articles and one review. The two studies dealt with the whole genome characterization of novel probiotic strains, one provided information related to the pro-technological and probiotic properties of several strains using predictive microbiology, while in the review article the currently available methods for assessing viability and stress tolerance of probiotics were presented.

Kiousi et al. characterized the probiotic and biotechnological potential of a *Lacticaseibacillus paracasei* strain, originally isolated from kefir grains by applying genomic tools. A chromosome map of this strain was built to determine its genomic stability, and then phylogenomic and comparative genomic analyses were applied to study the strain's metabolic capacity and ability to withstand environmental stresses. Several genes for heat, cold, osmotic shock, acidic pH, and bile salt tolerance were annotated, and it was revealed that the strain can utilize a plethora of carbohydrates as energy sources. Regarding the genome stability and safety, it was found that the strain does not harbor mobile elements, acquired antimicrobial resistance genes or virulence factors. Concerning the microbe-host interactions, adhesins, moonlighting proteins, exopolysaccharide biosynthesis genes were also pinpointed in the genome of *Lc. paracasei* SP5. The findings of this study suggested that *Lc. paracasei* SP5 is a good probiotic candidate with capacity to be incorporated in novel fermented food products.

Similarly, Tenea and Ascanta presented the genome sequencing and characterization of a novel *Lactiplantibacillus plantarum* strain isolated from wild naranjilla fruits. Safety of the strain was proven, since no acquired resistance genes nor virulence and pathogenic factors were predicted. Moreover, several molecular tools indicated the presence of multiple genes encoding for bacteriocins and ABC transporters and secondary metabolite regions which might confer a wide range of biotechnological benefits. The application of a targeted genome mining tool unraveled a diverse arsenal of important antimicrobial molecules such as lanthipeptides, where *in vitro* analysis revealed that the crude extract of the strain exerted a wide spectrum of inhibition against several pathogens. Furthermore, the antimicrobials produced by the strain are promising candidates to be tested *ex vitro* as biocontrol agents against foodborne pathogens during food processing and storage for the increasing of the shelf-life and safety of food products.

In another approach, Di Biase et al. studied the growth cardinal parameters of four *Lacticaseibacillus paracasei* strains and the acquired parameters were further used to simulate the growth of *Lc. paracasei* strains in cabbage and predict the time to reach the targeted probiotic level, using *in silico* simulations. Experiments *in vitro* and in mild processed white cabbage were performed to determine the effect of temperature and pH on the maximum specific growth rate of the selected strains. Maximum specific growth rates of *Lc. paracasei* IMPC2.1 in white cabbage were used to calculate the correction factor defined as the bias between the bacterial maximum specific growth rate in broth and in the food matrix. The findings of this study showed the potential of the predictive microbiology to foresee the growth of beneficial and pro-technological strains in foods to optimize the fermentative process.

Finally, Wendel reviewed the methods assessing viability and stress tolerance of probiotics. A probiotic product must be able to tolerate exposure to several stressors during the manufacturing process, storage, transportation, and through the passage in the human body and therefore be able to give a beneficial health effect. However, these stresses can trigger the transfer of culturable populations into non-culturable states, though these populations can be metabolically active. In that sense, culture-dependent and culture-independent methods for viability assessment were presented in her article. It was summarized that the disadvantage with methods based solely on culturability are their inability to detect all subpopulations that are included in the viable cell population. On the other hand she mentioned that the culture-independent molecular methods have the strength to identify and separate probiotics on a strain level but in this case the detection of viability marker is limited. Finally, the importance of choosing a holictic approach that reveals the whole picture of the activity of a probiotic was thoroughly presented.

In conclusion, the articles included in this Research Topic provided several examples of indigenous probiotic strains possessing desirable properties i.e., probiotic, antimicrobial, technological, adhesion capacity etc., *in vitro* and after whole genomic sequencing. However, the necessity of evaluation of the probiotic capacity of the strains concerning a real food ecosystem must be considered in future studies.

## Author contributions

All authors listed have made a substantial, direct, and intellectual contribution to the work and approved it for publication.
